# Activation of pro-survival autophagy by a small molecule promoting p62 oligomerization

**DOI:** 10.1016/j.jbc.2026.113430

**Published:** 2026-08-11

**Authors:** Johan Panek, Edward Fielder, Congsing Sun, Steph Crabtree, Tetsushi Kataura, Niall Wilson, Lakshana Baheerathan, Jiangyu Tang, Laura Booth, Wyatt Yue, Gavin Richardson, Lauren Holder, Gavin J. Miller, Jóhannes Reynisson, Sovan Sarkar, Viktor I. Korolchuk

**Affiliations:** 1 Biosciences Institute, Faculty of Medical Sciences, Newcastle University, Newcastle Upon Tyne, UK; 2 Department of Cancer and Genomic Sciences, School of Medical Sciences, College of Medicine and Health, University of Birmingham, Birmingham, UK; 3 Department of Neurology, Institute of Medicine, University of Tsukuba, Tsukuba, Japan; 4 School of Chemical and Physical Sciences and Glycoscience Centre, Keele University, Newcastle Under Lyme, UK; 5 School of Allied Health Professions and Pharmacy, Keele University, Newcastle Under Lyme, UK

**Keywords:** autophagy, Niemann-Pick type C1 disease, mitophagy, oligomerization, p62, ROS

## Abstract

Autophagy is a critical mechanism of cellular quality control, orchestrated by selective autophagy receptor (SAR) proteins. Pharmacologically enhancing the cargo-targeting capacity of SARs presents an attractive but underexplored strategy for the precise therapeutic activation of autophagy. Here, we characterize SQ-1, a small-molecule activator of autophagy that engages the prototypical SAR protein p62/sequestosome-1 (SQSTM1). We show that SQ-1 sensitizes p62 to oxidation and promotes its disulfide-mediated oligomerization in response to mitochondrial reactive oxygen species (ROS). This ROS-dependent activation of p62-mediated selective autophagy enhances the clearance of ROS-generating mitochondria and restores cell viability in models of Niemann-Pick type C1 disease, which is marked by impaired autophagic flux. In summary, the unique mode of action of SQ-1 enables self-regulated autophagy activation, offering a potential therapeutic strategy for lysosomal storage disorders and a broader spectrum of age-related diseases characterized by defective autophagy.

Macroautophagy (hereinafter autophagy) promotes proteostasis and longevity by degrading dysfunctional cellular components (1). This homeostatic role of autophagy requires dedicated receptor proteins recognizing damaged organelles and protein aggregates for the selective recruitment into autophagosomes, which deliver their cargo for degradation by lysosomes. p62 has been implicated in orchestrating several autophagy pathways by clustering ubiquitinated cargo and recruiting components of autophagy machinery for autophagosome assembly (2). Oligomerisation of p62 is an essential step in promoting the avidity of its interactions with autophagy proteins such as the lipidated forms of Atg8/LC3 incorporated into the nascent autophagosome (3, 4). The process of p62 oligomerization is mediated by electrostatic intermolecular interactions between its Phox and Bem1 (PB1) domains and can be further facilitated by the oxidation of two cysteine residues (Cys105 and Cys113) forming disulfide-linked conjugates (DLCs) (5, 6, 7). Non-covalent PB1 domain-mediated interactions and DLC have been shown to promote the ability of p62 to induce autophagic degradation of ubiquitinated proteins and mitochondria (mitophagy) (5, 8).

Disruption of selective autophagy has been implicated in various diseases including neurodegeneration, cancer, and lysosomal storage disorders, underscoring the need for targeted strategies to restore autophagic function (9). Several groups have recently developed small molecules targeting p62 and activating autophagy (8, 10, 11). These small molecules are designed to mimic the N-terminal arginine protein degradation signal that is recognized by ZZ-type zinc finger domain of p62. By binding to the ZZ domain, they facilitate the formation of DLC and increase autophagy flux (6, 7, 8). Our group has recently identified a novel p62-targeting small molecule STOCK1N which we re-named here to SQ-1 (a parent tool compound in “SeQuestra” series) (8). SQ-1 robustly promotes the mitophagic clearance of dysfunctional mitochondria. This can mitigate and even reverse the development of cellular aging phenotypes in primary human dermal fibroblasts (8).

At the same time, the molecular mechanisms by which p62-targeting small molecules promote DLC formation, selective autophagy, as well as their effect on cellular physiology remain poorly understood. It has been proposed that small molecule binding releases the interaction of the p62 ZZ domain with an unstructured linker region between PB1 and ZZ domains which contains Cys105 and Cys113 (11). This can potentially expose these residues to the oxidizing environment. Indeed, we demonstrated that oxidation-sensitive cysteine residues of p62 were essential for the ability of our p62-targeting small molecule to induce mitophagy (8). However, the origin and the role of localized reactive oxygen species (ROS) triggering disulfide bond and DLC formation in this process has not been explored.

Here we show that residues Asp129 and Arg139 in p62 are required for the activation of autophagy by SQ-1, indicating these as the site of its binding within the ZZ domain of p62. Furthermore, both PB1 domain- and DLC-mediated oligomerization of p62 are necessary for the autophagy-promoting effect of SQ-1 mediated by sensitization of p62 to oxidation by mitochondrial ROS. By using cell models of Niemann-Pick type C1 (NPC1) disease associated with loss of function of the lysosomal cholesterol transporter NPC1 along with defective autophagic flux, we demonstrate that SQ-1 can reactivate dysfunctional autophagy/mitophagy, reduce mitochondrial load characterized by high levels of ROS, and rescue cell death of NPC1 patient-derived fibroblasts and neurons. Together, our study provides a mechanistic underpinning for the development of pharmacological interventions promoting selective autophagy in the context of lysosomal storage disease and potentially relevant to other neurodegenerative disorders.

## Results

### SQ-1 activates general and selective autophagy

**Figure 1:**
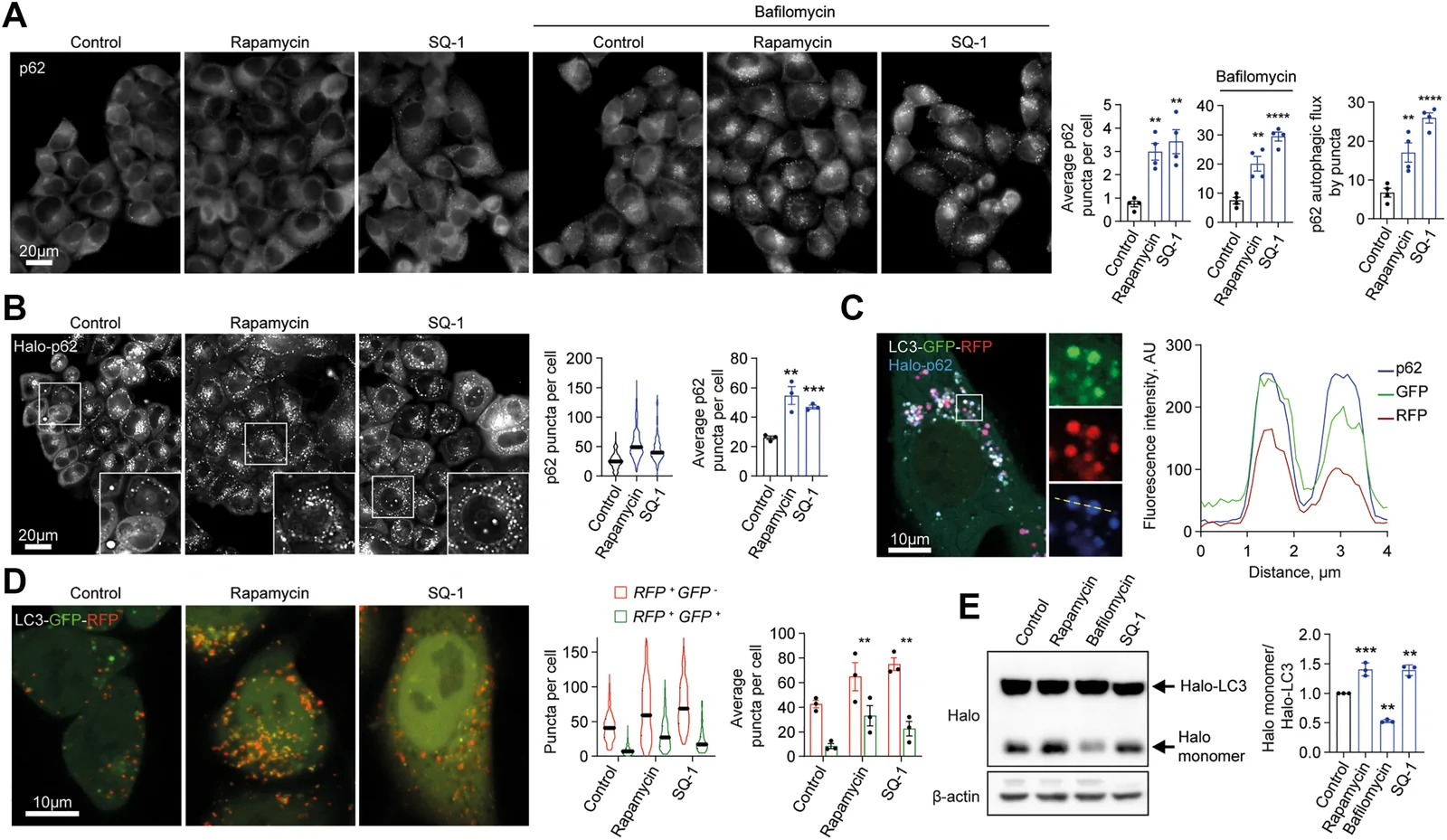
**Small molecule SQ-1 promotes inclusion of p62 into autophagosomes and activates autophagy.***A*, immuno-fluorescence microscopy images and quantification of p62 puncta and flux in wild-type HeLa cells treated with 50 nM rapamycin or 40 μM SQ-1 for 5 h, with and without 400 nM bafilomycin A1. *B*, fluorescence microscopy images and quantification of p62 puncta in PentaKO HeLa cells expressing Halo-p62 labeled with Halo ligand (Janelia Fluor 646) and treated with 50 nM rapamycin or 40 μM SQ-1 for 5 h. *C*, confocal microscopy images of PentaKO HeLa cells expressing mRFP-GFP-LC3 autophagy reporter and Halo-p62 labeled with Halo ligand and treated with 40 μM SQ-1 for 5 h. The plot is showing the intensity values for p62, GFP and RFP signal over the transect indicated by the *yellow line*. *D*, fluorescence microscopy images and quantification of autophagosomes and autolysosomes in PentaKO HeLa cells expressing mRFP-GFP-LC3 and Halo-p62 supplemented with 50 nM rapamycin or 10 μM SQ-1 for 24 h. *E*, immunoblot analyses and quantification of Halo monomer accumulation in HeLa cells expressing Halo-LC3 treated with 50 nM rapamycin, 400 nM bafilomycin A1, or 40 μM SQ-1 in the presence of HaloTag ligand for 5 h. Data are mean ± SEM of n = 3 biological replicates. *p* values were calculated by one-way ANOVA followed by multiple comparisons with Dunnett’s test (*A*, *C*, *E*, and *F*) or two-way ANOVA followed by multiple comparisons with Dunnett’s test (D). ∗*p* < 0.05; ∗∗*p* < 0.01; ∗∗∗*p* < 0.001; ns (nonsignificant). PentaKO, penta-knockout.

Our previous study employed primary human dermal fibroblasts, commonly used as the model of cellular senescence, to demonstrate that activation of autophagy and specifically mitophagy by SQ-1 can potently rescue cellular aging phenotypes (8). To facilitate identification of molecular mechanisms underpinning the action of SQ-1, here we initially investigated its effect in WT HeLa cells expressing endogenous levels of p62. Cells were treated for 5 h with 40 μM SQ-1 or 50 nM rapamycin, an inhibitor of mechanistic target Of rapamycin complex 1 and a potent autophagy inducer used as a control (12, 13), in the presence or absence of 400 nM bafilomycin A1. Both SQ-1 and rapamycin increased the formation of p62 puncta, p62 puncta flux, and LC3 flux (Figs. 1*A*, S1*A*). In contrast to rapamycin, the effect of SQ-1 was not mediated by the suppression of mechanistic target of rapamycin complex 1 activity (Fig. S1*B*) (12). Using an analog of SQ-1 that has poor predicted binding to p62 (termed SQ-Inactive, see Methods) did not induce p62 or LC3 puncta in WT HeLa cells (Fig. S1, *C*–*F*). To investigate the effect of SQ-1 on selective forms of autophagy and its reliance on p62, we employed HeLa CRISPR p62 WT and KO cells expressing a mitophagy reporter mt-mKeima (13, 14). Both SQ-1 and rapamycin induced mitophagy in WT cells, but only rapamycin induced mitophagy in p62 KO cells (Fig. S1, *G* and *H*). This indicated that SQ-1 is a potent activator of general and selective autophagy pathways and requires p62 for its action.

Next, we employed penta-knockout (PentaKO) HeLa cell line with the loss of 5 selective autophagy receptors (SARs) (p62, OPTN, NDP52, NBR1, and TAX1BP1) (14). Stable expression of transgenic p62 constructs in this cell line allowed us to further investigate p62-specific effects of SQ-1 in the absence of compounding factors mediated by the related SAR family members. To validate previous findings in this model, PentaKO cells expressing Halo-tagged WT p62 construct were labeled with Halo ligand (Janelia Fluor 646) and treated for 5 h with 40 μM SQ-1 or 50 nM rapamycin. Both compound treatments strongly increased the formation of p62 puncta, which, in the presence of the mRFP-GFP-LC3 autophagy reporter, colocalised with both GFP^+^ and RFP^+^ structures (Fig. 1, *B* and *C*) (15). This indicated that, similar to rapamycin, SQ-1 induces assembly of p62 into autophagic vesicles.

To compare the effect of SQ-1 and rapamycin on the activation of autophagic flux, mRFP-GFP-LC3 and Halo-p62 expressing PentaKO HeLa cells were treated with the compounds for 24 h and analyzed by fluorescence microscopy to quantify the number of autophagosomes (RFP^+^ and GFP^+^) and autolysosomes (RFP^+^ and GFP^-^) (12). Both treatments significantly increased the levels of early- and late-stage autophagic vesicles which demonstrate a strong induction of the entire autophagy pathway (Fig. 1*D*). An orthogonal immunoblotting assay using cells expressing Halo-LC3 reporter confirmed similar induction of autophagy flux in response to SQ-1 and rapamycin treatment, whilst inhibition of autophagy by bafilomycin A1 validated the specificity of the assay (Fig. 1*E*) (13). To investigate the effect of SQ-1 on mitophagy independent of other SARs, we employed PentaKO HeLa cells expressing Halo-p62 and mt-mKeima where mitophagy was strongly induced by both SQ-1 and rapamycin (Fig. S1*I*).

### SQ-1 engages p62 and activates autophagy in a p62-dependent manner

**Figure 2:**
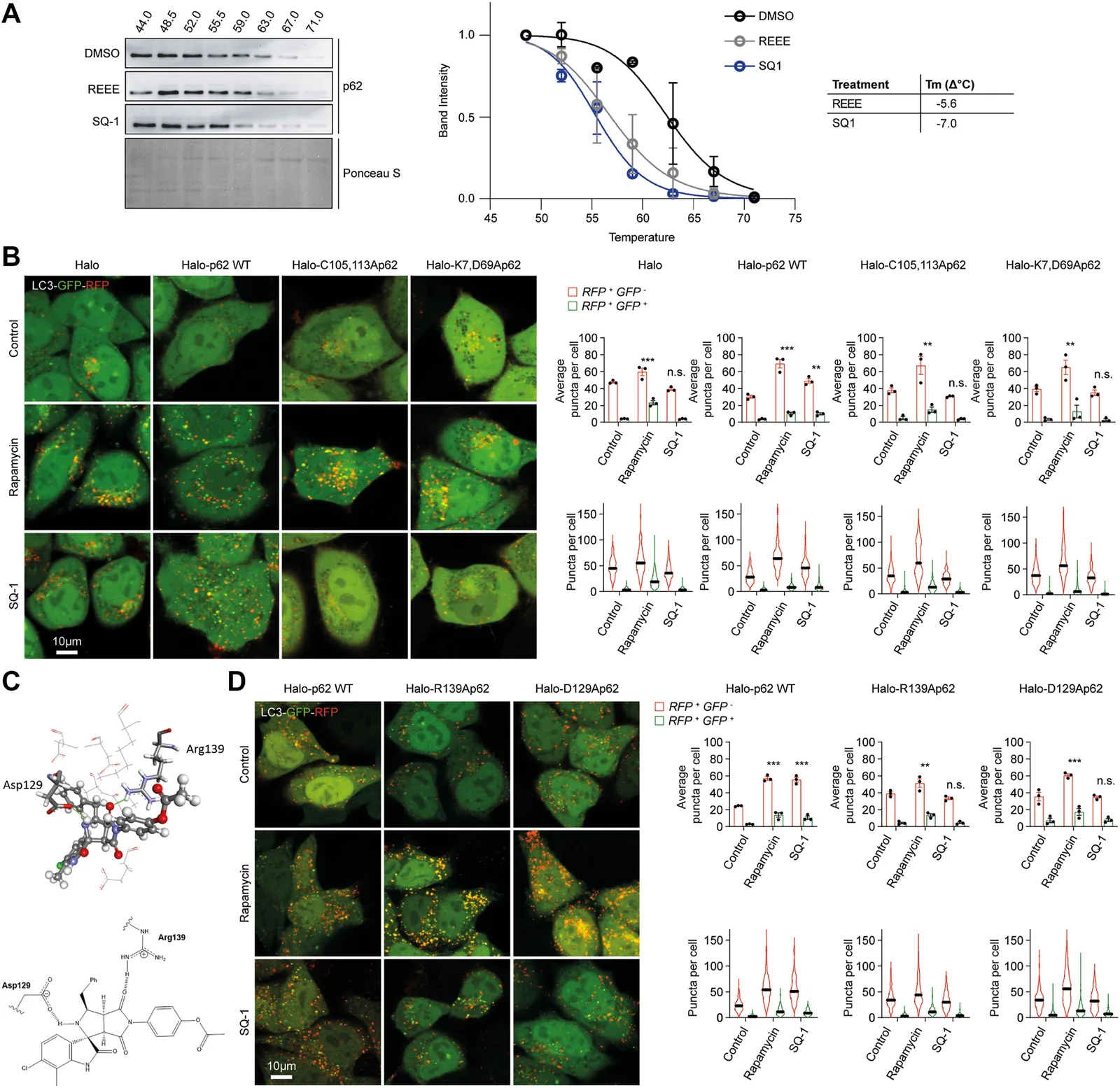
**SQ-1 induces autophagy in a p62 ZZ domain- and oligomerization-dependent manner.***A*, CETSA analysis of p62 following treatment with 100 μM SQ-1 or the 100 μM p62 ZZ-domain ligand REEEDK peptide (REEE) in cell lysates. Representative immunoblot showing soluble p62 remaining following thermal challenge across the indicated temperature range. Ponceau S staining is shown as an example of a loading control. Changes in melting temperature (ΔTm) relative to DMSO-treated controls are indicated. *B*, fluorescence microscopy images and quantification of autophagosomes and autolysosomes in PentaKO HeLa cells expressing mRFP-GFP-LC3 and Halo-p62 WT, Halo-C105A,C113Ap62 or Halo-K7A,D69Ap62 cultured in DMEM supplemented with 50 nM rapamycin or 10 μM SQ-1 for 24 h. *C*, the docked pose of SQ-1 (ball-and-stick format) in the binding site of p62 as predicted by the ASP scoring function. H-bonding is predicted between the carboxylic side chain of Asp129 and the pyrrolidine amine group of the central ring scaffold of the ligand (2.1 Å) as well as Arg139’s guanidine side chain with one of the carbonyl groups of the succinimide ring (1.7 Å). The adjacent amino acids (<5 Å), buttressing the ligand, are shown as *lines*. The amino acids’ hydrogens are not shown for clarity. Below is the two-dimensional structure of SQ-1 with the H-bonding interactions shown. *D*, fluorescence microscopy images and quantification of autophagosomes and autolysosomes in PentaKO HeLa cells expressing mRFP-GFP-LC3 and Halo-p62 WT, Halo-R139Ap62 or Halo-D129Ap62 cultured in DMEM supplemented with 50 nM rapamycin or 10 μM SQ-1 for 24 h. Data are mean ± SEM of n = 3 biological replicates. *p* values were calculated by two-way ANOVA followed by multiple comparisons with Dunnett’s test (*A*, *C*). ∗∗*p* < 0.01; ∗∗∗*p* < 0.001; ns (nonsignificant). ASP, Astex Statistical Potential; DMSO, dimethyl sulfoxide.

To investigate SQ-1 engagement with p62, we performed a cellular thermal shift assay (CETSA), a method of evaluating drug-target interactions (16). As a positive control, we used the REEEDK peptide, a previously described ligand of the p62 ZZ domain (11). Treatment with either SQ-1 or REEEDK altered the thermal stability profile of p62, resulting in a reduction in the apparent melting temperature of the protein (Fig. 2*A*). While CETSA cannot distinguish between direct ligand binding and ligand-induced conformational or protein-complex changes, these data are consistent with engagement of p62 by SQ-1 and provide independent support for the proposed interaction between the compound and p62.

To further test the requirement of p62 for autophagy activation, we used PentaKO HeLa cells expressing mRFP-GFP-LC3 in the absence or presence of Halo-p62 constructs to investigate the requirement for p62 during autophagy activation by SQ-1 and rapamycin. SQ-1 increased the formation of autophagosomes or autolysosomes in cells expressing WT Halo-tagged p62, but not empty Halo vector, indicating that p62 is both necessary and sufficient for the induction of autophagy flux in response to the small molecule (Fig. 2*B*). At the same time, rapamycin significantly increased autophagy in a p62-independent manner consistent with its established mechanism of autophagy activation (15). Cells expressing p62 mutants defective for DLC formation (C105A, C113A-p62) or PB1 domain oligomerization (K7A, D69A-p62), as well as cells expressing p62 mutants defective for ubiquitin binding (ΔUBA-p62) and LC3-interaction (ΔLIR-p62), phenocopied cells carrying empty Halo construct. In these cells, rapamycin, but not SQ-1, could activate autophagy (Figs. 2*B*, S2, *A* and *B*) (5, 8). Together, these data demonstrate that induction of autophagy by SQ-1 is strictly dependent on p62 and requires its ability to oligomerize, both *via* disulphide bonds and PB1 domain interactions. The requirement for both the UBA domain and the LIR motif suggests that SQ-1 does not bypass canonical p62 cargo recognition mechanisms. Rather, by promoting p62 oligomerization (8), SQ-1 is likely to increase the efficiency with which p62 engages preexisting ubiquitinated cargo and recruits autophagic membranes, thereby enhancing selective autophagic flux without necessarily increasing substrate ubiquitination (3).

To investigate potential off-target effects of SQ-1 on proteostasis pathways, we assessed proteasome function and lysosomal activity. SQ-1 did not affect the levels of the Ub^G76V^-GFP proteasome reporter (17) following either 5 h or 24 h treatment, indicating that it does not impact proteasomal degradation under the conditions used (Fig. S2, *C*–*E*). Similarly, SQ-1 did not alter LysoTracker accumulation within lysosomes, suggesting that lysosomal acidification was unaffected (Fig. S2*F*). Furthermore, SQ-1 did not impair the proteolytic maturation of cathepsins B or D, with precursor and mature species comparable to rapamycin-treated cells (Fig. S2*G*). Together, these findings indicate that SQ-1 does not exert its effects through nonspecific disruption of proteasome function, lysosomal acidification or lysosomal protease maturation, supporting a mechanism involving modulation of p62-dependent selective autophagy.

Our modeling of SQ-1 complex with ZZ domain of p62 indicated that, similar to previously published synthetic p62 ligands, amino acid residues Asp129 and Arg139 may become engaged in critical electrostatic interactions with the small molecule (Fig. 2*C*) (7, 11). p62 constructs carrying Ala substitutions of these residues were generated and expressed together with the mRFP-GFP-LC3 reporter in PentaKO HeLa. As shown in Figure 2*D*, Asp129 and Arg139 were found to be essential for the ability of SQ-1 (but not rapamycin) to induce autophagy flux. We conclude that binding of SQ-1 to the specific pocket within ZZ domain containing Asp129 and Arg139, as well as oligomerisation of p62, are essential elements of the mechanism underpinning the activation of autophagy by SQ-1. Together with the CETSA data, these findings support a model in which SQ-1 engages the ZZ domain of p62 through a pocket involving Asp129 and Arg139.

### Activation of autophagy by SQ-1 requires ROS

SQ-1, as well as p62-targeting small molecules developed by others, promote the formation of p62 DLC that are essential for the ability of SQ-1 to induce autophagy (7, 8). However, the mechanism by which the small molecules promote oxidation of redox-reactive cysteine residues triggering disulphide formation remains unknown. Our density functional theory (DFT) calculations indicated that SQ-1 is redox stable. The ionization potential (one-electron oxidation) of SQ-1 is 7.52 eV with 95% of drugs lying in the 6.0 to 9.0 eV range; the electron affinity (one-electron reduction) is −1.16 eV with drugs ranging from −1.5 to 2.0 eV (95% confidence interval) (18). SQ-1 is in the middle of the ionization range but relatively low for the electron affinity albeit still within the reported range of known drugs (Table S1). The low electron affinity can most easily be explained by the ester group occupying the *para* position on one of the phenyl rings (Fig. 2*C*). Therefore, oxidation of p62 is unlikely to be caused by SQ-1 directly.

**Figure 3:**
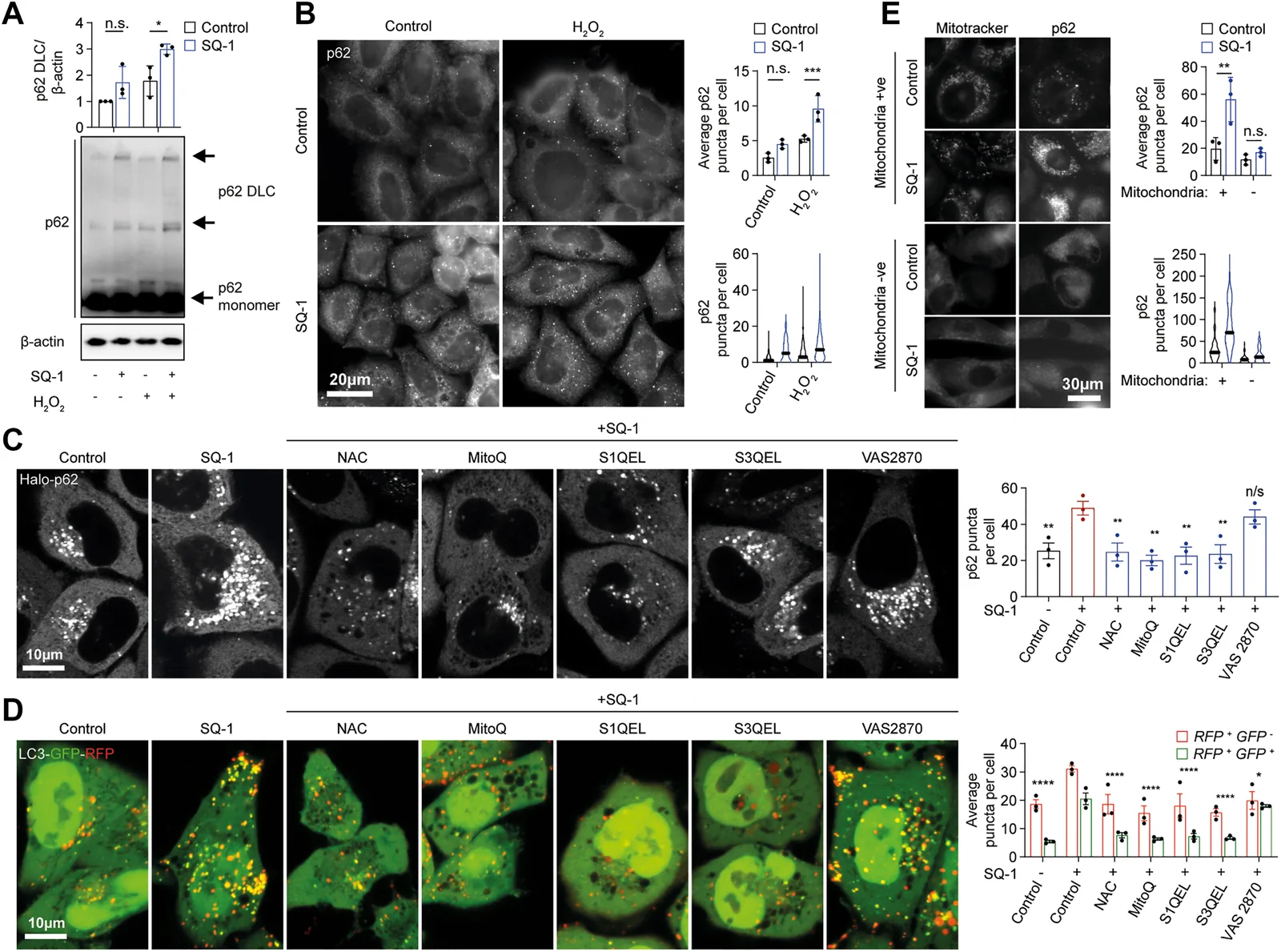
**SQ-1 induces autophagy by sensitizing p62 to oxidation by mitochondria-derived ROS.***A*, immunoblot analyses in nonreducing condition and quantification of p62 DLCs accumulation in HeLa cells treated with 30 μM SQ-1 for 18 h followed by H_2_O_2_ at 100 μM for 10 min. *B*, immunofluorescence microscopy images and quantification of p62 puncta accumulation in HeLa cells treated as in A. *C*, fluorescence microscopy images and quantification of p62 puncta in PentaKO HeLa cells expressing Halo-p62 labeled with Halo ligand (Janelia Fluor 646) and treated with 40 μM SQ-1, 1 mM NAC, 500 nM MitoQ, 10 μM S1QEL, 10 μM S3QEL, or 10 μM VAS 2870 for 5 h. *D*, confocal fluorescence microscopy images and quantification of autophagosomes and autolysosomes in HeLa cells expressing mRFP-GFP-LC3 treated as in C. *E*, fluorescence microscopy images and quantification of p62 puncta in mitochondria-retaining (mitochondria + ve) and mitochondria-deficient (mitochondria -ve) cells labeled with Halo ligand (Janelia Fluor 646) ± 40 μM SQ-1 in PentaKO HeLa cells expressing Halo-p62 (previously transiently transfected to overexpress YFP-Parkin and then treated twice during 45 h with 10 μM FCCP). Data are mean ± SEM of n = 3 biological replicates. *p* values were calculated by one-way ANOVA followed by multiple comparisons with Dunnett’s test (*C*) or two-way ANOVA followed by multiple comparisons with Dunnett’s (*D*) and Šidák’s test (*A*, *B*, and *E*). ∗*p* < 0.05; ∗∗*p* < 0.01; ∗∗∗*p* < 0.001; ns (nonsignificant). MitoQ, MitoQ10; NAC, N-acetyl-L-cysteine; ROS, reactive oxygen species.

We next investigated whether SQ-1 sensitizes p62 to oxidation by intracellular ROS, with a particular focus on mitochondria-derived ROS. We exposed WT HeLa cells to a short bout of low concentration hydrogen peroxide (100 μM, 10 min) following 18 h pretreatment of cells with SQ-1 or vehicle. Immunoblot analyses in nonreducing conditions, and immunofluorescence detection of intracellular puncta were used to ascertain the formation of DLC and self-assembly of p62, respectively (5, 8). SQ-1 pretreatment in the absence of exogenous H_2_O_2_ produced small but not statistically significant increase in DLC and p62 puncta formation (Fig. 3, *A* and *B*). This is in contrast to the data presented in Figure 1*A* where higher concentration was used in a shorter treatment. Importantly, in cells pretreated with SQ-1 the formation of p62 DLC and microscopically observable puncta was significantly higher when cells were given a short boost of hydrogen peroxide (Fig. 3, *A* and *B*). These data are consistent with the notion of p62 sensitization to oxidation by SQ-1 binding.

Next, we investigated whether endogenously generated ROS contribute to SQ-1-induced p62 oligomerization and sought to identify the principal intracellular source of these species. We tested several established chemical compounds: N-acetyl-L-cysteine (NAC) as a general antioxidant, mitochondria-targeted superoxide scavenger Mito Q10 (MitoQ), inhibitors of superoxide generation by mitochondrial complex I (S1QEL) or complex III (S3QEL), and an inhibitor of cytoplasmic NADPH oxidase VAS2870 (19, 20). Increased formation of p62 puncta, autophagosomes, and autolysosomes induced by SQ-1 treatment was completely suppressed by NAC or mitochondria-targeted molecules. In contrast, inhibitor of NADPH oxidase had little effect on SQ-1-induced p62 puncta formation and autophagosome/autolysosome numbers (Fig. 3, *C* and *D*). The effect of antioxidants was specific to SQ-1 response as formation of p62 puncta and autophagosome/autolysosome formation was not significantly affected in basal conditions (Fig. S3, *A* and *B*).

Given that mitochondrial-targeted, but not cytoplasmic NAPDH oxidase-targeted, antioxidants negated the SQ-1-induced increase in p62 puncta, we wanted to test whether mitochondria themselves were required for the effect of SQ-1. We transiently transfected p62 expressing PentaKO HeLa cells with YFP-Parkin and then induced mitophagy with high doses of carbonyl cyanide-p-trifluoromethoxyphenylhydrazone (FCCP), an established method to generate cells without mitochondria (21, 22). By doing this, we were able to deplete mitochondria from the transfected sub-population of cells (Fig. 3*E*). p62 puncta were predominantly present in cells retaining morphologically distinct mitochondria compared to mitochondria-depleted cells whilst SQ-1 treatment amplified p62 puncta formation specifically in cells retaining mitochondria (Fig. 3*E*). Together, these findings support a model in which physiological mitochondrial ROS generated during electron transport are a major contributor to SQ-1-induced p62 oligomerization and subsequent autophagy activation.

### SQ-1 rescues cellular deficits due to NPC1 deficiency

**Figure 4:**
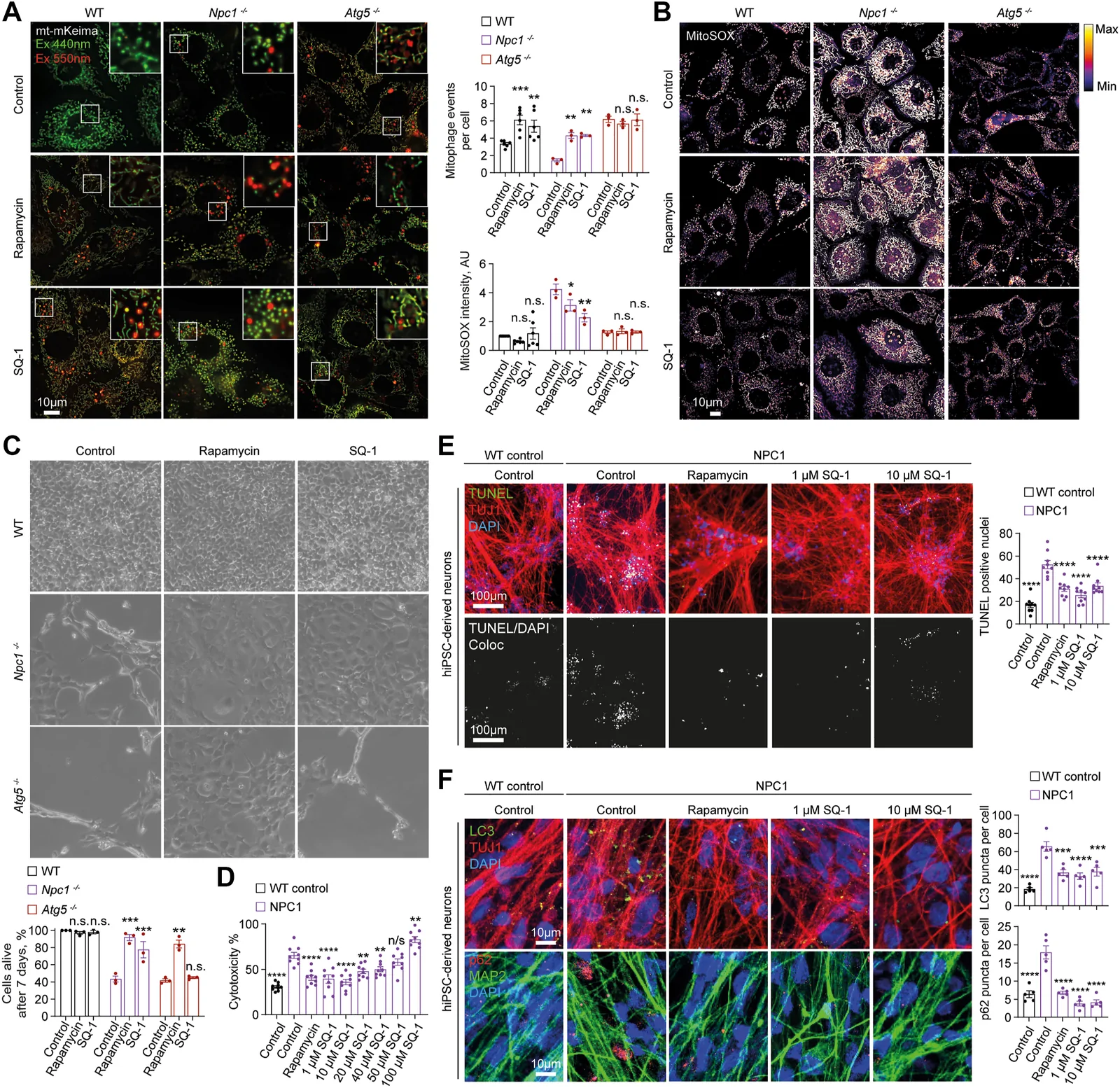
**SQ-1 rescues survival of NPC1 cells by activating autophagy/mitophagy.***A*, fluorescence microscopy images and quantification of mitophagy in wild-type (*Npc1*^*+/+*^ and *Atg5*^*+/+*^), *Npc1*^−/−^ and *Atg5*^*−/−*^ MEFs expressing mt-mKeima cultured for 24 h in galactose medium supplemented with 50 nM rapamycin or 10 μM SQ-1. *B*, fluorescence microscopy images and quantification of MitoSOX staining of wild-type (*Npc1*^*+/+*^ and *Atg5*^*+/+*^), *Npc1*^−/−^ and *Atg5*^*−/−*^ MEFs cultured for 24 h in galactose medium supplemented with 50 nM rapamycin or 10 μM SQ-1. *C*, phase-contrast images and cytotoxicity assay in *Npc1*^*+/+*^*, Npc1*^*−/−*^ and *Atg5*^*−/−*^ MEFs cultured in galactose medium supplemented with 50 nM rapamycin or 10 μM SQ-1 for 72 h (phase contrast image) or 144 h (cytotoxicity assay). *D*, cytotoxicity assay in control and NPC1 patient iPSC-derived cortical neurons after 4 weeks of neuronal differentiation, where neurons were treated with 50 nM rapamycin or 1 to 100 μM SQ-1 for 6 days. *E*, fluorescence images and quantification of TUNEL^+^ apoptotic nuclei in TUJ1^+^ cells treated as in D. *F*, fluorescence images and quantification of LC3B and p62 puncta in TUJ1^+^ or MAP2^+^ cells treated as in D. Data are mean ± SEM of n = 3 biological replicates. *p* values were calculated by one-way ANOVA followed by multiple comparisons with Dunnett’s test (*A*) or two-way ANOVA followed by multiple comparisons with Dunnett’s test (*B*, *C*, and *D*). ∗*p* < 0.05; ∗∗*p* < 0.01; ∗∗∗*p* < 0.001; ns (nonsignificant). iPSC, induced pluripotent stem cell; MEF, mouse embryonic fibroblast; NPC1, Niemann-Pick type C1.

Autophagy flux is disrupted in cellular models of NPC1 disease (12, 23, 24). In actively respiring cells with the loss/mutation of NPC1 protein, such as mouse embryonic fibroblasts (MEFs) cultured in galactose medium, or upon differentiation of patient-derived induced pluripotent stem cells (iPSCs) into neurons, the loss of mitochondrial quality control by autophagy results in an increased production of ROS and cell death (12, 19). Culturing cells in galactose medium increases reliance on mitochondrial oxidative phosphorylation and limits compensation by glycolytic ATP production, this unmasks disease-associated autophagic and mitochondrial dysfunction and the cell death phenotype of NPC cells (12). Using these models, we have shown previously that autophagy inducers, including rapamycin, celecoxib, and memantine, can restore autophagy/mitophagy flux in NPC1 deficient cells, reduce accumulation of mitochondria with high ROS levels, and rescue cell survival (12, 23). Similar to rapamycin, SQ-1 treatment resulted in the rescue of these phenotypes in *Npc1*^*−/−*^ MEFs cultured in galactose medium (Figs. 4, *A* and *B*, S4*A*) (12). However, unlike rapamycin which rescues cell death in the absence of autophagy (12), SQ-1 did not restore survival of autophagy-deficient *Atg5*^*−/−*^ MEFs cultured in galactose medium (Fig. 4*C*). These data indicate that the effect of SQ-1 on cell survival is strictly dependent on autophagy.

Next, we utilized NPC1 patient iPSC-derived neuronal cells, which are disease-relevant cellular platforms widely used to evaluate the efficacy of candidate drugs. NPC1 neurons exhibit increased cell death alongside a block in autophagic flux, involving accumulation of autophagosomes and p62, compared to the control neurons (12, 19, 25). We tested a range of SQ-1 concentrations and found that doses between 1 and 20 μM resulted in the rescue of cell death similar to rapamycin. At higher concentrations the cytoprotective effect was abolished, likely due to compound cytotoxicity within our extended treatment protocol (Fig. 4*D*). We therefore selected the lowest two concentrations (1 and 10 μM) to further investigate their effect on autophagy and cell death in neurons. Assessment of TUNEL staining for apoptotic nuclei in TUJ1-positive neuronal cells indicated that SQ-1, similar to rapamycin, effectively rescued cell death of NPC1 neurons (Fig. 4*E*) (12, 19). This cytoprotective effect was associated with reduced accumulation of autophagosomal markers LC3 and p62 in NPC1 neurons (Fig. 4*F*), consistent with a restoration of autophagic flux (12).

**Figure 5:**
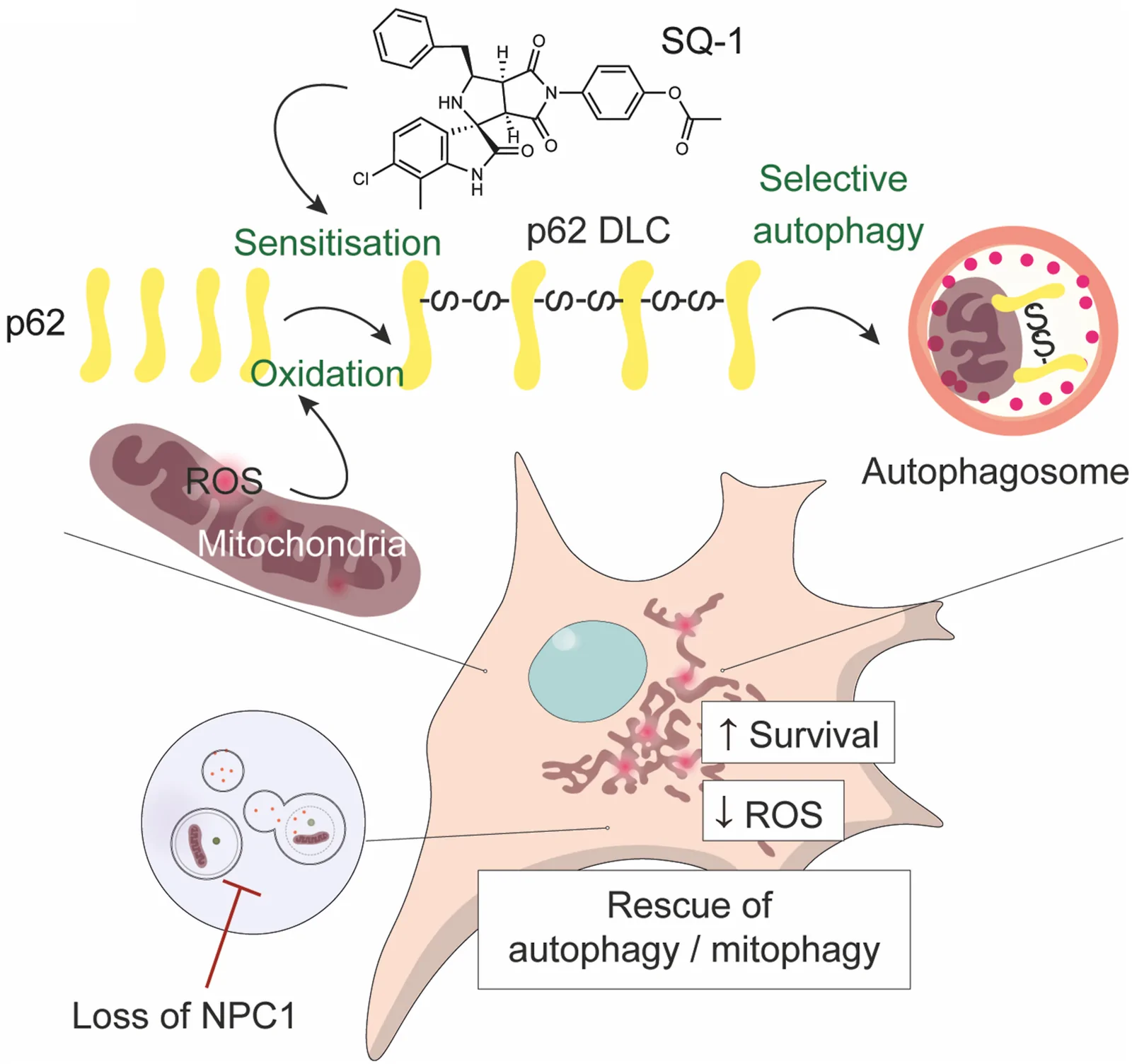
**Restoration of cellular quality control results in rescue of cell death caused by NPC1 deficiency**. Schematic representation of the proposed mechanism by which SQ-1 sensitizes p62 to mitochondria-generated ROS, leading to p62 oligomer formation, selective autophagy/mitophagy rescue, reduction in mitochondria with elevated ROS levels, and rescue of cell death. NPC1, Niemann-Pick type C1; ROS, reactive oxygen species.

Based on these data, we propose that by sensitizing p62 to oxidation by mitochondria-derived ROS, SQ-1 enhances the flux through p62-dependent selective autophagy pathway. This reduces the build-up of autophagosomes and accumulation of damaged cellular components, including mitochondria, characterized by elevated ROS levels in cells with NPC1 deficiency. Ultimately, the restoration of cellular quality control mechanisms results in the rescue of cell death caused by NPC1 deficiency (Fig. 5).

## Discussion

Selective autophagy acts as an important quality control mechanism by eliminating dysfunctional cellular components (1). As such, pharmacologically enhancing the activity of selective autophagy pathways may potentially offer novel therapeutic opportunities in a broad range of human diseases associated with an accumulation of damaged organelles such as mitochondria generating high levels of ROS. Targeting SARs, the molecular machines that recognize the cargo and directly interact with the core autophagy machinery, presents a unique approach to upregulating selective autophagy. By targeting p62, we can induce autophagy in a more selective manner than through targeting signaling pathways. This is in contrast to the majority of established autophagy activators, which modulate signaling cascades upstream and inevitably result in pleiotropic effects on cell biology (12, 26).

Our current study provides a mechanistic insight into the mode of action of p62-targeting ligands such as SQ-1. The small molecule appears to sensitize p62 to physiological ROS signals, such as from mitochondria, thereby activating autophagy. It can be hypothesized that by sensitizing p62 to the ROS signal and promoting its disulfide formation and oligomerization, SQ-1 facilitates targeting of the potentially damaged mitochondrial component for p62-dependent mitophagy. This model is supported by the observation that SQ-1-induced autophagy was abolished by general and mitochondrial-targeted antioxidants but was largely unaffected by inhibition of NADPH oxidase activity. One open question is the exact source and the nature of ROS promoting p62 DLC formation. The most likely explanation is that superoxide produced by mitochondrial complex I and III is dismutated into H_2_O_2_ which permeates into cytoplasm where it oxidizes p62 (20). However, the communication of mitochondria with the autophagy machinery is likely to be complex and dependent on different mitochondrial states. For example, mitochondrial depolarization and increased permeability associated with damage may allow for the leakage of superoxide into cytoplasm (20). Instead, high levels of carbon dioxide and ROS produced by metabolically active mitochondria will result in the formation of peroxymonocarbonate, a potent oxidizing agent which may similarly act as a localized cue for p62 DLC formation, albeit in response to the different mitochondrial state (27).

It will be particularly important to investigate oligomerization of p62 and the activation of autophagosome formation at the sub-mitochondrial level, in order to understand specific characteristics of mitochondria being targeted by p62 dependent mitophagy in response to SQ-1 treatment. The same arguments apply to the mechanisms by which p62 oligomerization/DLC formation may play the role in selective autophagy pathways other than mitophagy. Another important question pertaining to the mechanism of the p62-targeting small molecules is how engagement of the ZZ domain increases the protein’s propensity to disulfide formation. Recently a p62 ligand dusquetide has been proposed to bind two ZZ domains in the trans manner, effectively acting as a molecular staple bringing together two p62 monomers (10). However, our modeling studies did not identify potential binding of SQ-1 with the second p62 ZZ domain moiety. Instead, the model where binding of the ligand releases the linker region containing redox-regulated cysteine residues from its interaction with the ZZ domain thus allowing for the formation of intermolecular disulfide bonds appears to be the most likely hypothesis (11). Overall, CETSA analysis together with site-directed mutagenesis of the p62 ZZ domain supports a model in which SQ-1 engages a region of the ZZ domain involving Asp129 and Arg139, and thereby promotes p62 oligomerization and inducing autophagy in a p62-dependent manner. However, this model would require experimental testing, including the rigorous investigation of the direct binding of SQ-1 to p62 using structural biology and biophysics approaches as part of the effort to develop p62-targeting ligands into drug-like entities.

Importantly, our current data provide strong evidence for the cytoprotective effect of SQ-1 in cell models of NPC1, including patient iPSC-derived neurons. These data set the groundwork for future medicinal chemistry work and *in vivo* testing of p62-specific ligands. However, should these efforts be successful, it is clear that pharmacological interventions meaningfully improving cellular quality control mechanisms could be of significant and broad relevance to medicine, from rare forms of lysosomal storage diseases to slowing down the aging process itself.

## Experimental procedures

### Culture of mammalian cell models

Immortalized *Npc1*^+/+^ and *Npc1*^*−/−*^ MEFs (gift from Peter Lobel); *Atg5*^*+/+*^ and *Atg5*^*−/−*^ MEFs (gift from Noboru Mizushima) (28); WT and PentaKO HeLa cells (14) were maintained in DMEM supplemented with 10% fetal bovine serum (FBS), 100 U/ml penicillin/streptomycin, 2 mM L-glutamine (all from Sigma-Aldrich) at 37 °C, and 5% CO_2_ in a humidified incubator. 293FT cells (R70007, Invitrogen) were cultured as above in medium supplemented with 1 X MEM nonessential amino acids (Gibco).

Compound treatment conditions were optimized for the specific biological endpoint under investigation. Acute p62 oligomerization and puncta formation were assessed using shorter treatment periods, whereas autophagic flux and mitophagy assays employed longer incubations to allow sufficient time for cargo recognition, autophagosome maturation and lysosomal processing. Concentrations were selected based on preliminary assay optimization to maximize signal while maintaining cell viability throughout the experimental period. Treatment duration and concentration is provided in each relevant subsection below.

### iPSC culture and neuronal differentiation

Human iPSC lines, control and NPC1 iPSC lines were cultured as previously described (25, 29). The human iPSC lines were cultured on inactivated MEF feeder layer in hESC medium comprised of DMEM/F-12, 5% KnockOut Serum Replacement, 1% L-glutamine, 1% nonessential amino acids, 1% penicillin/streptomycin, 4 ng/ml human recombinant basic fibroblast growth factor (all from Gibco), 15% FBS (HyClone) and 0.1 mM β-mercaptoethanol (Sigma-Aldrich) and maintained in a humidified incubator with 5% CO_2_ and 5% O_2_ at 37 °C. For experimentation, the iPSCs were cultured feeder-free on Geltrex basement membrane matrix in StemFlex Basal Medium supplemented with StemFlex 10X Supplement (all from Gibco).

Neural stem cells (NSCs) were differentiated from iPSCs, as described previously (30, 31). NSCs were cultured on poly-L-ornithine and laminin (Sigma-Aldrich) coated plates or flasks in N2B27 medium comprised of DMEM/F-12 and Neurobasal medium in 1:1 ratio, 1% N-2 supplement, 2% B-27 supplement, 1% penicillin/streptomycin (all from Gibco), 0.1% β-mercaptoethanol (Sigma-Aldrich) supplemented with 10 ng/ml FGF-2 (Miltenyi Biotec), and 10 ng/ml EGF (PeproTech), and were maintained in a humidified incubator with 5% CO_2_ at 37 °C. NSCs were passaged twice a week with 0.05% Trypsin-EDTA (Gibco), and the medium was changed on alternate days.

Neuronal differentiation of human iPSC-derived NSCs was carried out as described previously (30, 31). The NSCs were seeded as above on poly-L-ornithine and laminin coated plates in N2B27 medium without FGF-2 and EGF. At day 4 of neuronal differentiation, cells were treated with 10 μM DAPT (Tocris) to prevent cell proliferation. The N2B27 medium (without FGF-2 and EGF) was changed every 2 days and neuronal differentiation was carried out for 4 weeks. The neurons generated *in vitro* were cortical in nature (30, 31).

The control and NPC1 patient-derived iPSCs were originally generated in the lab of Rudolf Jaenisch at the Whitehead Institute for Biomedical Research. These cell lines were used for this study in the lab of Sovan Sarkar at the University of Birmingham under material transfer agreements, UBMTA 15-0593 and UBMTA 15-0594. All experiments were performed in accordance with ISSCR and institutional guidelines and regulations.

### Generation of stable cell lines

Generation of cells stably expressing YFP-Parkin, mt-mKeima, Halo-LC3, Halo-GFP-KDEL, mRFP-GFP-LC3, or the Halo-p62 WT and mutant (K7A,D69A-p62, C105A,C113A-p62, D129A-p62, R139A-p62, M404V(ΔUBA)-p62, and ΔLIR-p62) constructs were achieved by packaging retroviruses or lentiviruses in the HEK293FT (293FT) cell line. Cells were seeded in a 10 cm dish (6.0 × 10^6^ cells/10 ml/dish) in antibiotic-free culture medium. The next day, cells were transfected with plasmids containing the packaging gag/pol (retrovirus) or psPAX2 (lentivirus) and envelope pCMV-VSV-G genes, and the YFP-Parkin-IRES-zeo (gift from Douglas Green and Stephen Tait, Addgene, 61728) (32), pCHAC-mt-mKeima (gift from Richard Youle, Addgene, 72342) (14), pMRX-IP-HaloTag7-LC3 and pMRX-IB-HaloTag7-mGFP-KDEL (gift from Noboru Mizushima, Addgene, 184901) (13) or Halo-p62 constructs (ordered from Vector Builders) using Lipofectamine 3000. Following overnight transfection, the medium was replaced with fresh antibiotic-free medium. After 48 h virus-containing medium was collected and concentrated using LentiX concentrator reagent (Takara Bio). The different cell lines were then transduced using concentrated virus and selected after 24 h using the appropriate selection antibiotic: Cells expressing mRFP-GFP-LC3, Halo-LC3 and the different Halo-p62 mutants were selected using 8 μg/ml of puromycin (Invitrogen). Cells expressing Halo-GFP-KDEL were selected using 4 μg/ml of blasticidin. Following selection, PentaKO HeLa cells transduced with Halo-p62 mutant constructs were labeled with Janelia Fluor 646 Halo ligand and sorted by fluorescence-activated cell sorting (FACS, BD FACSAria Fusion) to select cells with p62 expression level matching WT Halo-p62. To generate cell lines expressing both Halo-p62 mutant construct and mRFP-GFP-LC3 reporter, previously sorted cells were transduced a second time with mRFP-GFP-LC3 and GFP expressing cells were selected by FACS (BD FACSAria Fusion).

### Generation of p62 KO HeLa cell lines

HeLa cells were seeded at 2.5 × 10^5^ cells per well in a 6-well plate and transfected the following day using Lipofectamine 3000 (Thermo Fisher Scientific) according to the manufacturer's instructions. The transfection mixture contained an hCas9 expression plasmid (Addgene), an sgRNA construct targeting sequestosome-1 (SQSTM1)/p62 (target sequence: 5′-GGCGCCTCCTGAGCACACGG-3′), and pEGFP-C1 as a transfection reporter. The sgRNA constructs were sourced from the plasmid set described in (14). Forty-eight hours posttransfection, GFP-positive cells were isolated by FACS using a BD FACSAria Fusion cell sorter, and single cells were deposited into individual wells of a 96-well plate for clonal expansion.

Surviving clones were screened for loss of p62 expression by Western blot. Protein lysates were resolved by SDS-PAGE and immunoblotted with an anti-p62 antibody (Progen, Cat# GP62-C; RRID: AB_2687531). Clones lacking detectable p62 protein were expanded and verified by independent immunoblotting.

### p62 puncta formation assay

PentaKO HeLa cells transduced with either WT or mutant Halo tagged p62 were seeded into 24-well glass bottom plates (Greiner). 24 h later, cells were labeled with 40 nM Janelia Fluor 646 Halo ligand (GA1120, Promega) and then incubated for 5 h with following compounds: 50 nM rapamycin (R8781-200UL, Sigma-Aldrich), 400 nM bafilomycin A1 (BML-CM110-0100, Enzo Life Sciences), 40 μM SQ-1 (STOCK1N-57534, InterBioScreen Ltd), 1 mM NAC (A7250, Sigma-Aldrich), 500 nM MitoQ10 (S8978-SEL, Stratech Scientific Ltd), 10 μM S1QEL (F2068-0013, Life Chemicals), 10 μM S3QEL (SML1554, Sigma-Aldrich), and 10 μM VAS 2870 (BML-EI395-0010, Enzo Life Sciences). Live cells were then imaged on an inverted DMi8 microscope (Leica) with a Plan-Apochromat 63 x/1.40 oil immersion lens. The number of puncta per cell was quantified by outlining single cells as regions of interest using Cellpose software (33) and counting particles in cells using Analyze Particles plugin in Fiji/ImageJ. At least 50 cells per condition were quantified. For the colocalization between p62 puncta and LC3-GFP-RFP reporter, live cells were imaged on a LSM800 confocal microscope (Zeiss) and fluorescence intensity across a line was extracted using Fiji and plotted in Prism 10.1.2 software (GraphPad).

### Mitochondrial clearance

PentaKO HeLa cells transduced with WT p62-Halo were transiently transfected with YFP-Parkin, leading to a mixed population of cells expressing YFP-Parkin or not. Mitochondria were cleared from YFP-Parkin expressing cells as in (21) with 10 μM FCCP, substituting CCCP. Briefly, transfection mix contained a 1:1 ratio of YFP-Parkin and blank pcDNA in OptiMEM with Lipofectamine 2000 (11668019, Invitrogen). YFP-Parkin was a gift from Richard Youle (Addgene plasmid # 23955 (34)). Transfection mix was added for 5 h before being removed and washed with normal media. 24 h posttransfection, cells were treated with two sequential 24-h treatments of 10 μM FCCP, the media changed again and 48 h later, cells were treated with Halo ligand (Janelia Fluor 646) ± 40 μM SQ-1 for 5 h. This was removed and cells treated with 100 nM Mitotracker Green for 30 min, with 5 μM Hoechst 33,342 added after 15 min. Live cells were imaged using an inverted DMi8 microscope (Leica) with a Plan-Apochromat 63x/1.40 oil immersion lens.

### Assessment of autophagy

For immunoblot analysis, HeLa cells expressing Halo-LC3 and *Npc*^+/+^ and *Npc1*^*−/−*^ MEFs expressing Halo-GFP-LC3 were treated with 50 nM rapamycin, 400 nM bafilomycin A1, 40 μM (HeLa) or 15 μM (MEFs) SQ-1, and 20 μM 7-Bromo-1-heptanol (HaloTag-blocking agent) (H54762, Thermo Fisher Scientific Chemicals) (35) for 5 h (HeLa) or 24 h (MEFs), followed by immunoblot analysis using Halo antibody. Autophagic activity was quantified as a ratio of Halo monomer to Halo-LC3 or Halo-GFP-LC3.

For autophagy flux assays using mRFP-GFP-LC3 reporter, cells expressing the reporter were seeded in 35 mm glass bottom dishes and treated for 24 h with 50 nM rapamycin, 400 nM bafilomycin A1, and 10 μM SQ-1. For autophagy flux assays in the presence of antioxidants, cells were treated with 40 μM SQ-1 1 mM NAC, 500 nM MitoQ10, 10 μM S1QEL, 10 μM S3QEL, or 10 μM VAS 2870 for 5 h before imaging. Cells were imaged on a LSM700 confocal microscope (Zeiss) and the number of autophagosomes (GFP^+^ RFP^+^ puncta) and autolysosomes (GFP^-^ RFP^+^ puncta) per cell were quantified by outlining single cells as regions of interest using Cellpose software (33) and counting particles in cells using Analyze Particles plugin in Fiji/ImageJ. At least 50 cells per condition were quantified.

### H_2_O_2_ treatments

HeLa cells were seeded in 6-well plates (p62 Western blot) or on glass coverslips in 24-well plates (p62 immunofluorescence). After 24 h cells were treated with 30 μM SQ-1 for 18 h followed by H_2_O_2_ at 100 μM or 500 μM for 10 min. Cells were either fixed with 4% PFA for immunofluorescence or protein was extracted for p62 DLCs immunoblots as described below.

### Immunoblot analyses

HeLa and MEFs cells were seeded in 6-well 24 h prior to treatments. After treatments, cells were washed in ice-cold 1 × PBS then lysed in RIPA buffer (150 mM NaCl, 1% NP40, 0.5% NaDoC, 0.1% SDS, 50 mM Tris pH 7.4, supplemented with 1 × Halt Protease & Phosphatase inhibitor cocktail (Thermo Fisher Scientific) in ddH_2_O) plus 50 mM N-ethylmaleimide. Cell lysates were then centrifuged at 4 °C at 16,000*g* for 10 min to remove insoluble cellular components. Protein concentration was measured by the DC Protein Assay (BioRad) and a FLUOstar Omega plate reader (BMG Labtech). Samples were prepared by boiling in SDS Loading buffer (BioRad) at 100 °C for 5 min in the presence or absence of 2.5% β-ME (β-mercaptoethanol, Sigma-Aldrich). Amounts equivalent to 20 to 40 μg of protein were run on 10 to 12% Tris-Glycine SDS-PAGE gels and transferred to Immobilon-P (Millipore) polyvinylidene difluoride membranes using a Trans-Blot SD Semi-Dry Transfer Cell (BioRad). Blots were incubated with a blocking solution (PBS containing 5% fat-free dry milk, 0.1% Tween-20) for 1 h. After washing with PBS, blots were incubated with primary antibodies diluted in blocking solution at 4 °C overnight. Blots were then washed three times for 5 min in PBS with 0.1% Tween-20, incubated with secondary HRP-conjugated α-mouse or α-rabbit (#A2554 and #A0545, Sigma-Aldrich, 1:5000) or α-guinea pig (ab102360, Abcam, 1:1000) antibodies in blocking solution for 45 min at RT. Blots were washed three times and incubated for 5 min with the Clarity Western ECL Substrate (BioRad) and signals were detected by chemiluminescence using an iBright Imaging Systems (Thermo Fisher Scientific). The following primary antibodies were used: Mouse α-Halo (#G9211, Promega, 1:1000), mouse α-β-actin (STJ96930, St John’s Laboratory, 1:5000), guinea pig α-p62 (#GP62-C, Progen Biotechnik, 1:1000), rabbit α-S6 (2217S, CST, 1:1000), rabbit α-Phospho-p70 S6 Kinase (Thr389) (9205S, CST, 1:1000), mouse α-cathepsin B (C0715 Sigma-Aldrich, 1:200), mouse α-cathepsin D (C6243, Sigma-Aldrich, 1:200), and rabbit α-GFP (A6455, Invitrogen, 1:2000). Where blots are quantified, densitometry using area under the curve was performed in Fiji/ImageJ (version 1.54i; NIH), normalized against the specified house-keeping product and then to the control.

### Mitophagy assay

To enforce mitochondrial respiration, HeLa and MEFs expressing YFP-Parkin and mt-mKeima were cultured for 24 h in galactose medium (glucose-free DMEM (Gibco) supplemented with 10 mM D-galactose, 10 mM Hepes, 1 mM sodium pyruvate, 4 mM L-glutamine, 100 U/ml penicillin/streptomycin and 10% FBS (all from Sigma-Aldrich). After 24 h, galactose medium was supplemented with 100 nM rapamycin or 10 μM SQ-1 for 24 h. Live-cell mt-mKeima signal was obtained using a DMi8 microscope (Leica) and mitophagy events were determined *via* following steps using Fiji/ImageJ (version 1.54i; NIH) (20). Images were masked by applying MaxEntropy threshold algorithm to the images obtained with 561 nm excitation to remove low red signal and background. Within the masks, signals of mt-mKeima were adjusted by applying Enhanced Contrast plugin with saturated = 0.1, normalize, and equalize options. Then, images were generated by subtracting the signal at a 480 nm excitation (reporting neutral pH-environment) from the signal at a 561 nm excitation (reporting an acidic pH-environment). Resulting images were binarized with the MaxEntropy threshold algorithm to extract mitolysosomes. The number of puncta per cell was quantified by outlining single cells as regions of interest using Cellpose software (33) and counted using Analyze Particles plugin in Fiji/ImageJ.

### Immunofluorescence

Fixed cells were permeabilized in methanol for 4 min at −20 C and then blocked for 1 h in 5% normal goat serum (Sigma-Aldrich) in PBS at RT. Blocked coverslips were incubated with antibody (mouse α-p62 (#610833, BD Sciences, 1:200), for p62/LC3 costaining rabbit α-p62 (#PM045, MBL, 1:500) and mouse α-LC3 (ALX-803-081, Enzo) overnight at 4 C. Cells were washed three times and incubated with goat secondary antibody (α-mouse Alexa Fluor 594 secondary antibody (A-11005, Thermo Fisher Scientific, 1:1000), and for p62/LC3 costaining addition of α-rabbit Alex Fluor 488 (#A-11034, Thermo Fisher Scientific, 1:1000)) for 1 h at RT. Cells were washed, and coverslips were mounted on slides with Prolong gold antifade with DAPI mounting medium (#P36931, Invitrogen). Fluorescence images were obtained on a Dmi8 microscope (Leica). The number of puncta per cell in images was quantified by outlining single cells as regions of interest using Cellpose software (33) and counted using Analyze Particles plugin in Fiji/ImageJ.

iPSC-derived cortical neurons were washed in PBS, fixed with 4% formaldehyde at RT for 15 minutes, permeabilized with 0.5% Triton X-100 for 10 minutes (except LC3B staining) or with prechilled methanol for 5 minutes (for LC3B antibody), and incubated with Blocking buffer (5% goat or donkey serum (Sigma-Aldrich) in PBS with or without (LC3B staining) 0.05% Tween 20 for 1 hour at RT. Cells were then incubated overnight with primary antibodies at 4 °C, followed by incubation with Alexa Fluor conjugated secondary antibodies for rabbit (A21206, Thermo Fisher Scientific) or mouse (A21203, Thermo Fisher Scientific) for 1 hour at RT. The coverslips were mounted on glass slides with ProLong Gold antifade reagent with DAPI (Invitrogen). The following primary antibodies were used: rabbit α-LC3B (NB100-2220, Novus Biologicals, 1:200), mouse α-p62 (610832, BD Biosciences, 1:200), mouse α-TUJ1 (4466, CST, 1:200), and rabbit α-MAP2 (8707, CST, 1:200).

### Mitochondrial ROS measurement

*Npc*^+/+^ and *Npc1*^*−/−*^ MEFs seeded in a 35 mm glass bottom dishes were transferred to galactose medium 24 h post seeding. The next day, galactose medium was supplemented with 50 nM rapamycin or 10 μM SQ-1 for 24 h, then stained with 2.5 μM MitoSOX (M36008, Invitrogen) for 10 minutes, washed three times with galactose medium and imaged on a Dmi8 microscope (Leica). Fluorescence intensity was analyzed by outlining single cells as regions of interest and calculation of the integrated density value per cell. The resulting values were then normalized using WT MEFs MitoSOX intensity as a baseline.

### Proteosome and cathepsin assessment

pUb^G76V^ GFP HeLa cells (gift from Nico Dantuma, Karolinska Institute) (17) were seeded in 24 well glass bottom plates. Cells were treated for 5 and 24 h, with 50 nM rapamycin, 40 μM for 5 h or 10 μM for 24 h SQ-1, 5 μM of Lactacystin or 10 μM of MG-132. Cells were stained with 5 μM Hoechst 33,342 for 15 min and washed 3 times with media. Live cells were imaged using an inverted DMi8 microscope (Leica) with a Plan-Apochromat 40x oil immersion lens. Puncta per cell were counted in cells using Analyze Particles plugin in Fiji/ImageJ. For immunoblot analysis, cells were seeded in a 6-well plate and treated as above and harvested as described under “Immunoblot analysis”.

### Lysotracker assay

WT HeLa cells were seeded on cell culture slides (10 chambers, 543979, Greiner Bio-One) and treated for 5 and 24 h, with 50 nM rapamycin, (40 μM for 5 h and 10 μM for 24 h) SQ-1 or 50 nM bafilomycin A1. Cells were stained with 50 nM lysotracker red in media for 30 min, with 5 μM Hoescht added at 15 min. Live cells were imaged using an inverted DMi8 microscope (Leica) with a Plan-Apochromat 40 x oil immersion lens. Fluorescence intensity was analyzed by outlining single cells as regions of interest and calculation of the integrated density value per cell.

### Cytotoxicity assays

Cytotoxicity in WT, *Npc1*^*−/−*^, *Atg5*^*−/−*^ MEFs was measured using Cytotox-Glo Cytotoxicity Assay (G9291, Promega) according to manufacturer instruction. Cells were seeded in a 96-well white plate (Greiner) and switched to galactose medium after 24 h. After 144 h in galactose medium, cells were incubated with Cytotox-Glo Assay Reagent for 15 min at RT in the dark, then luminescence was measured using a GloMax plate-reader (Promega) and the readings obtained were attributed to the basal cytotoxicity per well (first reading). To estimate cell population per well, cells were further incubated with lysis reagent for 30 min at RT in the dark, after which luminescence was measured again (second reading). Population of cell alive was then calculated by subtracting the first reading (basal cytotoxicity per well) to the second reading (indicative of cell population per well) and normalized to the WT control condition (dimethyl sulfide (DMSO)).

TUNEL staining for apoptotic neuronal cells were performed using Click-iT Plus TUNEL Assay kit (C10617, Invitrogen), according to the manufacturer’s protocol. For detection of TUNEL^+^ apoptotic nuclei specifically in neurons, cells were subjected to immunofluorescence by blocking with 3% BSA (in PBS) followed by incubation with anti-TUJ1 antibody (801201, BioLegend, 1:1000) overnight at 4 °C, and thereafter incubated with Alexa Fluor 594 secondary antibody for 1 hour at RT. Coverslips were mounted on glass slides with ProLong Gold antifade reagent with DAPI (Invitrogen). The quantification of TUNEL^+^ apoptotic nuclei in TUJ1^+^ neuronal cells was performed *via* fluorescence microscopy, as previously described (25). The percentage of TUNEL^+^ nuclei was calculated from the total number of TUJ1^+^ cells analyzed.

### Modeling of SQ-1-p62 binding

The SQ-1 and the negative control STOCK1N-58141 (SQ-Inactive) ligands were docked to the binding site of the p62/SQSTM1 ZZ domain (PDB ID: 5YP8, resolution 1.45 Å) (36). The scoring functions GoldScore(GS) (37), ChemScore(CS) (38, 39), Piecewise Linear Potential (ChemPLP) (40) and Astex Statistical Potential (41) were used in the GOLD (v2024.1) docking algorithm. The docking centre for the binding pocket was defined as the side chain carbonyl carbon of Asp129 (x = 8.242, y = −14.427, z = 197.93) with a radius of 10 Å; this is the same docking site as was used in the initial virtual screen leading to the discovery of SQ-1 (8). Fifty docking runs were allowed for each ligand with default search efficiency (100%). The basic amino acids lysine and arginine were defined as protonated. Furthermore, aspartic and glutamic acids were assumed to be deprotonated.

The results for the scoring functions are given in Table S1 in the SI. Overall SQ-1 has better predicted binding as compared to SQ-Inactive with the expectation of Astex Statistical Potential, which is slightly higher. Consensus docking was used to identify SQ-1 in the initial virtual screen (8), *i.e.*, the four scoring functions predicted a similar binding pose. Consensus docking is an established approach to increase the reliability of pose predictions of docking algorithms (42, 43). None of the scoring functions predicted the same pose for SQ-Inactive indicating poor binding to the p62/SQSTM1 ZZ domain (see Fig. S5, *A* and *B*).

### DFT calculations

The Gaussian 16 software suite was used with unrestricted DFT. The B3LYP functional hybrid approach was employed (44, 45) and standard 6-31G(d,p) basis set (46, 47) was used for geometry optimization and frequency analysis (keywords: opt freq). The zero-point vibrational energies were scaled according to Wong (0.9804) (48). In all cases, normal modes revealed no imaginary frequencies indicating that they represent minima on the potential energy surface. The subsequent energy calculations were then performed with the larger 6-311G(2df, p) basis set. Adiabatic ionization potentials and adiabatic electron affinities were calculated as described (49). The calculated energies and zero-point vibrational energies are given in Table S2.

### Cell thermal shift assay (CETSA)

WT HeLa cells were seeded onto 10 cm dishes and incubated for 24 h at 37 C. Cells were collected in ice-cold PBS supplemented with Halt Protease and Phosphatase Inhibitor Cocktail (Thermo Fisher Scientific, 78430) and centrifuged at 500*g* for 3 min at 4 C. Cell pellets were resuspended in ice-cold lysis buffer (100 mM Tris, pH 7.5, 150 mM NaCl, 0.2% NP-40, and 2 x Halt Protease and Phosphatase Inhibitor Cocktail) and subjected to three freeze-thaw cycles using liquid nitrogen. Lysates were centrifuged (10 min, 16,100*g* at 4 C) to pellet insoluble material. Soluble supernatants were collected and incubated with either DMSO vehicle, 100 μM SQ-1 or 100 μM REEEDK peptide for 1 h on ice. DMSO content was balanced for all conditions. Following compound incubation, lysates were aliquoted into individual PCR tubes and subjected to a thermal gradient (44, 48.5, 52, 55.5, 59, 63, 67, and 72 C) for 3 min using a PCR thermocycler. Samples were again centrifuged (10 min, 16,100*g* at 4 C) to pellet thermally denatured proteins. The soluble supernatants were collected for protein quantification, normalization, and immunoblotting analysis.

### Structure and purity verification

#### HPLC analysis

SQ-1 was dissolved in acetonitrile (10 mg/ml) and analytical HPLC was performed using Agilent 1220 Infinity LC equipped with a variable wavelength detector and Agilent Infinity LC/MS mass spectrometer, on a reverse phase column (Poroshell 120Å EC-C18. 4.6 × 100 mm, 2.7 μm). Flow rate 0.3 ml/min, Solvent A: water + 0.1% formic acid, Solvent B: acetonitrile, column equilibrated with 5%B, 10 min to 95%B, held at 95%B for 4 min, 1 min to 5%B then held at 5% for 5 min. Injection volume 1 × 10 μl, injection concentration (10 mg/ml) and UV detection at 260 nm. The HPLC-C18-UV-MS spectrum is given in Fig. S5, *C* and *D* and purity of SQ-1 in Table S3 as 96.94%.

#### NMR analysis

^1^H NMR spectra were recorded using a Brüker Magnet system 400′54 Ascend (400 MHz) at RT in CDCl_3_ as a reference. Chemical shifts are given as δ in parts per million (ppm) and all peaks are relative to tetramethylsilane (TMS) where δ TMS = 0.00 ppm. Each signal has a multiplicity given by the following abbreviations: s (singlet), br s (broad singlet), d (doublet), dd (doublet of doublets), t (triplet), and m (multiplet). Also, integrations of peaks are given by nH, where n is the number of protons for that resonance. Where appropriate, coupling constants are measured in Hertz (Hz) and have been stated to the nearest 0.1 Hz. ^13^C NMR spectra were recorded using a Brücker Magnet system 400′54 Ascend (100 MHz) at RT in CDCl_3_ as a reference. PENDANT sequence was used as well as an internal deuterium lock. Each peak of the spectra is given as δ in ppm and all peaks are relative to TMS where δ TMS = 0.00 ppm. The NMR spectra of SQ-1 is given in Fig. S5, *E*–*H* and assignments are as follows.

**Figure:**
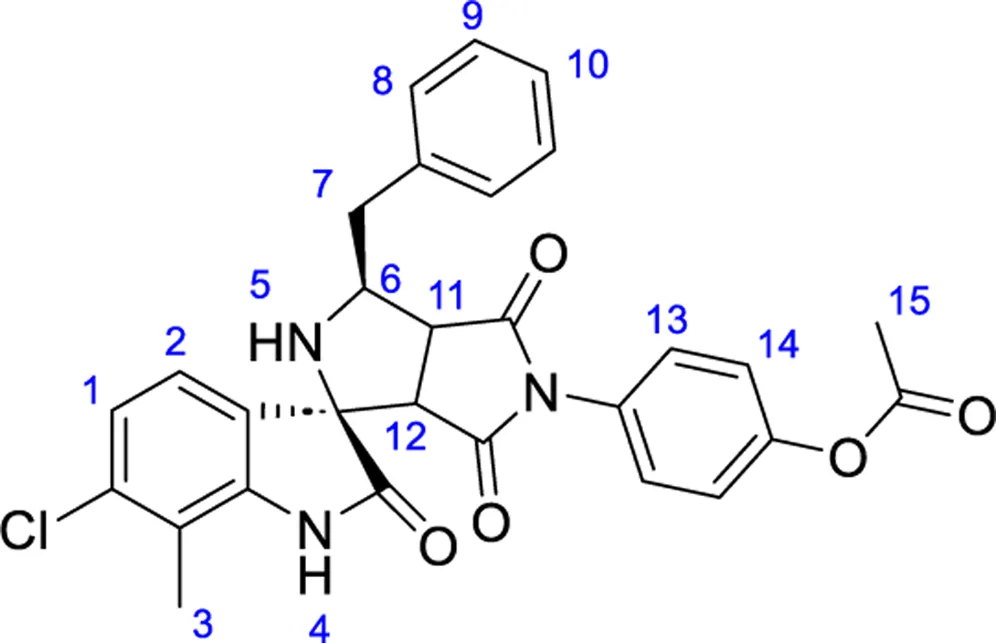

^1^H NMR (400 MHz, CDCl_3_) δ: 8.15 (s, 1H, *H*-4/*H*-5), 7.48 – 7.40 (m, 2H, *H*-8/*H*-13/*H*-14), 7.31 – 7.23 (m, 6H, *H-*8/*H-*13/*H-*14, *H*-9), 7.23 – 7.15 (m, 1H, *H*-10), 7.04 (d, *J* = 7.9 Hz, 1H, *H*-1), 6.82 (d, *J* = 7.9 Hz, 1H, *H*-2), 4.72 (ddd, *J* = 11.0, 7.5, 3.9 Hz, 1H, *H*-6), 3.80 (d, *J* = 7.9 Hz, 1H, *H*-12), 3.75 – 3.67 (t, *J* = 7.5 Hz, 1H, *H*-11), 3.47 (dd, *J* = 13.8, 3.9 Hz, 1H, *H*-7a), 2.76 (dd, *J* = 13.8, 10.1 Hz, 1H, *H*-7b), 2.33 (s, 3H, *H*-15), 1.96 (s, 3H, *H*-3).

**Figure:**
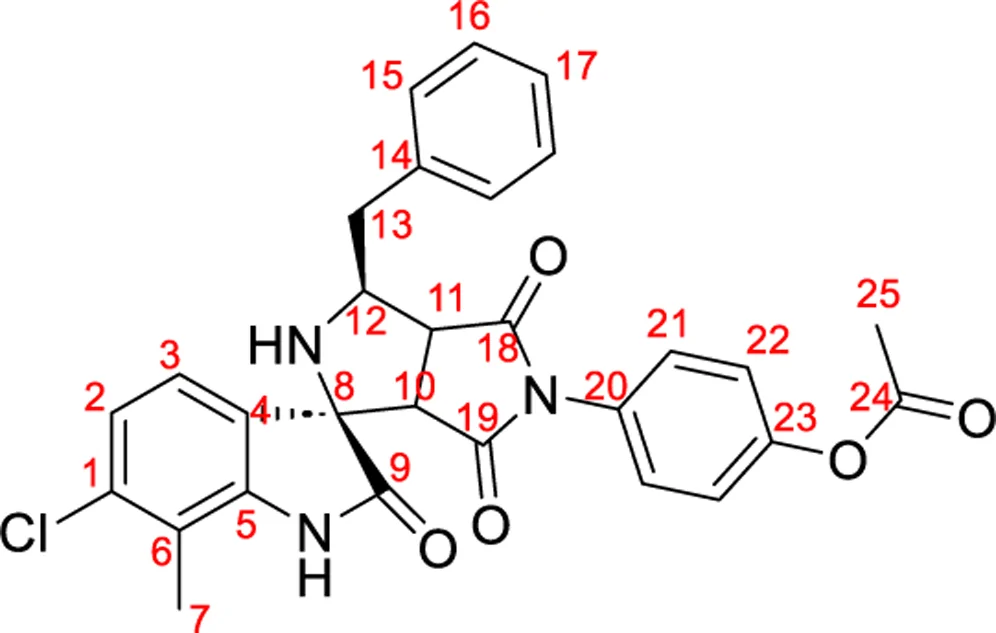

^13^C NMR (100 MHz, CDCl_3_) δ: 180.5 (*C*-9/*C*-18/*C*-19/*C*-24), 175.0 (*C*-9/*C*-18/*C*-19/*C*-24), 174.4 (*C*-9/*C*-18/*C*-19/*C*-24), 169.0 (*C*-9/*C*-18/*C*-19/*C*-24), 150.6 (*C-*1/*C-*4/*C-*5/*C-*6/C-14/*C-*20/*C*-23), 140.5 (*C-*1/*C-*4/*C-*5/*C-*6/C-14/*C-*20/*C*-23), 139.1 (*C-*1/*C-*4/*C-*5/*C-*6/C-14/*C-*20/*C*-23), 136.2 (*C-*1/*C-*4/*C-*5/*C-*6/C-14/*C-*20/*C*-23), 129.1 (*C-*1/*C-*4/*C-*5/*C-*6/*C*-14/*C-*20/*C*-23), 128.9 (*C-*1/*C-*4/*C-*5/*C-*6/*C*-14/*C-*20/*C*-23), 128.7 (*C-*1/*C-*4/*C-*5/*C-*6/*C*-14/*C-*20/*C*-23), 127.3 (*C-*15/*C*-21/*C*-22), 126.6 (*C*-17), 124.5 (*C*-3), 123.5 (*C*-2), 122.5 (*C-*15/*C-*16/*C-*21/*C-*22), 68.1 (*C*-8), 59.0 (*C*-12), 51.6 (*C*-10), 47.6 (*C*-11), 37.8 (*C*-13), 21.2 (*C*-25), 13.4 (*C*-7).

### Statistical analysis

All experiments were carried out in three or more biological replicates from independent cell culture. Graphical data denote the mean ± SEM (of n = 3 biological replicates) and are depicted by column graph scatter dot plot, using Prism 10.1.2 software (GraphPad). Unless indicated otherwise, the *p* values was determined by one-way or two-way ANOVA followed by multiple comparisons using Dunnett’s or Šidák’s statistical hypothesis testing using Prism 10.1.2-10.4.2 software (GraphPad).

## Data availability

Original images of immunoblotting are provided as supplementary material. All other datasets generated in this study are presented and analyzed within this article and are available from the corresponding author upon request.

## Supporting information

This article contains supporting information.

## Conflict of interest

V. I. K. is a Scientific Advisor for Longaevus Technologies. S. S. is a Scientific Advisor for NMN Bio Ltd. All other authors declare they have no competing interests.
