# Supplementary material for: Activation of pro-survival autophagy by a small molecule promoting p62 oligomerization
Source: J Biol Chem. 2026 Aug 11;302(10):113430. doi: 10.1016/j.jbc.2026.113430 (PMC13570303; doi:10.1016/j.jbc.2026.113430)
Supplement: Supplementary file 1 [file mmc1.docx]

**Supplementary Figure 1. Small molecule SQ-1 promotes autophagy and mitophagy.**

**A**, Immunoblot analysis and quantification of p62 and LC3 in wild-type HeLa cells treated with 50 nM rapamycin or 40 µM SQ-1 for 5 h, with and without 400 nM bafilomycin A1. **B**, Immunoblot analyses and quantification of S6 and phospho-S6 in HeLa cells treated with 50 nM rapamycin or 10 µM SQ-1 for 24 h. **C, D,** Immuno-fluorescence microscopy images and quantification of p62 (C) and LC3 (D) puncta in wild-type HeLa cells expressing p62 and treated with 50 nM rapamycin, 40 µM SQ-1 or 40 µM SQ-Inactive for 5 h. **E, F,** Quantification of p62 and LC3 puncta in C and D, respectively. **G, H**, Fluorescence microscopy images and quantification of mitophagy in HeLa CRISPR p62 wild-type (G) and p62 knock-out (H) cells, expressing mt-mKeima reporter cultured in galactose (representative images shown) or DMEM medium supplemented with 50 nM rapamycin or 10 µM SQ-1 for 24 h. **I**, Fluorescence microscopy images and quantification of mitophagy in PentaKO HeLa cells expressing Halo-p62 and mt-mKeima reporter cultured in galactose medium supplemented with 50 nM rapamycin or 10 µM SQ-1 for 24 h. Data are mean ± SEM of n = 3-4 biological replicates. P values were calculated by one-way ANOVA followed by multiple comparisons with Dunnett’s test. *P< 0.05; **P<0.01; ***P<0.001; ****P<0.0001; ns (non-significant).

**Supplementary Figure 2. SQ-1 activates autophagy in p62 ubiquitin and LC3 binding-dependent manner.**

**A**, Fluorescence microscopy images and **B,** quantification of autophagosomes and autolysosomes in PentaKO HeLa cells expressing mRFP-GFP-LC3 and Halo-p62 WT, Halo-ΔUBA-p62, Halo-ΔLIR-p62 cultured in DMEM supplemented with 50 nM rapamycin or 10 µM SQ-1 for 24 h. **C**, Fluorescence microscopy images and **D,** quantification of ubiquitin^G67V^-GFP puncta in wild-type HeLa cultured in DMEM supplemented with 50 nM rapamycin, 40 µM SQ-1 or 5 µM lactacystin for 5 h and 50 nM rapamycin, 10 µM SQ-1 or 5 µM lactacystin for 24 h. **E,** immunoblots of ubiquitin^G67V^-GFP following 24 hours treatment with 50 nM rapamycin, 10 µM SQ-1, 5 µM lactacystin or 10 µM MG-132 for 24 h. **F,** Fluorescence microscopy images of lysotracker and quantification of lysotracker signal per lysosome in wild-type HeLa cultured in DMEM supplemented with 50 nM rapamycin, 40 µM SQ-1 or 5 µM lactacystin for 5 h and 50 nM rapamycin, 10 µM SQ-1 or 5 µM lactacystin for 24 h. **G,** immunoblots of LAMP1, cathepsin B and cathepsin D in wild-type HeLa cultured in DMEM supplemented with 50 nM rapamycin, 40 µM SQ-1, 5 µM lactacystin, 10 µM MG-132 or 400 nM bafilomycin for 5 h, or 50 nM rapamycin, 10 µM SQ-1 or 400 nM bafilomycin for 24 h. Data are mean ± SD of n = 3 biological replicates. P values were calculated by two-way ANOVA followed by multiple comparisons with Šidák’s test (B). *P< 0.05; **P<0.01; ***P<0.001; ****P<0.0001; ns (non-significant).

**Supplementary Figure 3. Antioxidants do not significantly affect basal autophagy.**

**A**, Confocal fluorescence microscopy images and quantification of p62 puncta in PentaKO HeLa cells expressing Halo-p62 labelled with Halo ligand (Janelia Fluor 646) and treated with 1 mM N-Acetyl-L-cysteine, 500 nM MitoQ10, 10 µM S1QEL, 10 µM S3QEL or 10 µM VAS 2870 for 5 h. Note that control images from Figure 3C and D are used for comparison. **B**, Confocal fluorescence microscopy images and quantification of autophagosomes and autolysosomes in HeLa cells expressing mRFP-GFP tandem fluorescent-tagged LC3 cultured in DMEM supplemented with 1 mM N-Acetyl-L-cysteine, 500 nM MitoQ10, 10 µM S1QEL, 10 µM S3QEL or 10 µM VAS 2870 for 5 h. Data are mean ± SEM of n = 3 biological replicates. P values were calculated by one-way ANOVA followed by multiple comparisons with Dunnett’s test (A) or two-way ANOVA followed by multiple comparisons with Šidák’s test (B). *P< 0.05; **P<0.01; ***P<0.001; ns (non-significant).

**Supplementary Figure 4. SQ-1 ameliorates autophagy deficit in *Npc1^-/-^* MEFs.**

**A**, Immunoblot analyses and quantification of Halo monomer accumulation in *Npc1*^+/+^ and *Npc1*^-/-^ MEFs expressing Halo-LC3 cultured for 24 h in galactose medium supplemented with 50 nM rapamycin or 15 µM SQ-1. Data are mean ± SEM of n = 3 biological replicates. P values were calculated by one-way ANOVA followed by multiple comparisons with Dunnett’s test. *P< 0.05; **P<0.01; ***P<0.001; ns (non-significant).

**Supplementary Figure 5. HPLC-C18-UV-MS and NMR analysis of SQ-1.**

**A,** The structure of the negative control STOCK1N-58141. **B**, The different poses predicted by the different scoring functions (ASP – green, ChemPLP – red, CS – blue and GS – element). **C**, HPLC-C18-UV-MS detection trace at 260 nm. **D**, Mass spectrum for peak at 8.908 min; Theoretical [M]^−^ for SQ-1 m/z = 529.98. **E,** ^1^H NMR spectrum of SQ-1 in CDCl_3_. **F,** HSQC NMR spectrum of SQ-1 in CDCl_3_. **G,** COSY NMR spectrum of SQ-1 in CDCl_3_. **H,** ^13^C NMR spectrum of SQ-1 in CDCl_3_.

**Table S1**. The binding affinities as predicted by the scoring functions and their corresponding H-bonding strength.

| **Nr.** | **Catalogue** | **ASP** | **ChemPLP** | | **CS** | **GS** |
| --- | --- | --- | --- | --- | --- | --- |
| **SQ-1** | STOCK1N−57534 | 21.8 | 58.9 | 21.8 | | 53.6 |
| **6a** | STOCK1N−58141 | 24.4 | 43.4 | 14.5 | | 50.6 |

**Table S2**. The single point and corrected zero-point vibrational energies (ZPE) of SQ-1 in hartrees (a.u.).

| **Nr.** | **Systems** | **Energy/a.u.** | **ZPE/a.u. ^a^** |
| --- | --- | --- | --- |
| **Parent** | SQ-1 | -2120.714688 | 0.471317496 |
|  | IP | -2120.436381 | 0.469237087 |
|  | EA | -2120.751507 | 0.465686078 |

^a^ Corrected according to [52].

**Table S3.** HPLC-C18-UV-MS peak area analysis of SQ-1, detection at 260 nm. Purity calculated as 96.94 %.

| **Retention time (min)** | **Width (min)** | **Peak Area** | **Peak Height** | **Area%/ %** |
| --- | --- | --- | --- | --- |
| **7.881** | 0.0555 | 254.5533 | 76.4727 | 1.9002 |
| **8.630** | 0.0525 | 155.0530 | 49.2524 | 1.1575 |
| **8.908** | 0.0696 | 12986.1875 | 3108.1707 | **96.9423** |
|  | **Sum** | 13395.7938 |  |  |
